# Landing control algorithm for gimbal-serviced UAVs based on field-of-view constraints

**DOI:** 10.1038/s41598-025-20044-3

**Published:** 2025-10-15

**Authors:** Wenlong Mao, Zhanxiang Li, Mingen Huo

**Affiliations:** 1 Present Address: Unmanned Systems Research Center, China People’s Police University, Guangzhou, 510000 China; 2 Present Address: School of Automation Science and Engineering, South China University of Technology, Guangzhou, 510641 China

**Keywords:** Autonomous UAV landing, Visual servo control, Field-of-view constraint, Prescribed performance control, Velocity estimation, Engineering, Mathematics and computing

## Abstract

This paper presents a robust and adaptive visual servoing-based landing control method for unmanned aerial vehicles (UAVs) equipped with a three-axis gimbal camera. To address the limitations of fixed-camera configurations, the proposed approach integrates pixel-level field-of-view (FOV) constraints and leverages the gimbal’s agility for enhanced visual tracking. The landing task is formulated as a constrained image-based control problem, where tracking errors of image features are rigorously bounded using prescribed performance functions. A velocity observer is incorporated to estimate the time-varying motion of the landing platform in real time, enabling accurate autonomous landing without relying on external communication or infrastructure. Lyapunov-based stability analysis confirms the theoretical soundness of the control strategy. Simulation results validate the effectiveness and robustness of the proposed method, demonstrating improved accuracy, adaptability, and practical applicability in UAV landing scenarios.

## Introduction

Multi-rotor unmanned aerial vehicles (UAVs), valued for their flexibility, maneuverability, and ease of deployment^1,2^, have become essential tools for performing complex tasks in both military^3^ and civilian domains^4^. This has prompted a wide range of research concerning related UAV systems^5,6^ and their control architectures^7–9^. However, their high energy consumption and limited flight endurance constrain their operational capabilities^10^. The development of safe and reliable autonomous landing technologies^11^ enables UAVs to efficiently interface with dedicated landing platforms that provide energy replenishment and maintenance support, thereby significantly extending flight endurance and enhancing overall mission efficiency.

Vision-based autonomous landing control methods^1^, known for their high precision, low cost, and ease of implementation^12^, have shown strong potential in delivering reliable landing information in complex and unstructured environments. Consequently, considerable research efforts have been devoted to UAV landing control^13,14^.

To address underactuation and external disturbances in UAV systems, an adaptive robust control algorithm with dynamic compensation^15^ was proposed. This approach achieves precise position and attitude control during both horizontal and vertical tracking maneuvers, enabling stable landing operations in complex environments. Furthermore, a novel helical docking mechanism^16^ facilitates autonomous UAV-UGV separation and docking through collaborative information sharing, significantly enhancing the terrain adaptability of heterogeneous systems. Ship hull motion estimation via onboard cameras and nonlinear prediction^17^ enabled the decomposition of unmanned surface vessel (USV) periodic movement. This facilitated the implementation of a model predictive control (MPC) strategy for autonomous UAV landings on maritime platforms. Separately, an invariant ellipsoid theory-based control design^18^ quantified platform motion and environmental disturbances (e.g., wind, noise), enabling optimal gain tuning for smooth landing operations. An autonomous landing framework integrating GPS with vision-based navigation^19^ employs a visual guidance pipeline incorporating convex hull transformation, interference exclusion, ellipse fitting, and feature matching. Separately, a UAV-surface vessel cooperative landing system^20^ utilizes multi-ultrasonic joint localization and hierarchical guidance point generation to achieve stable recovery on dynamic maritime platforms. An adaptive tracking control scheme integrating backstepping with dynamic surface control^21^ guides UAVs to target-centered safe zones using monocular downward-facing vision, enabling precision landings. Concurrently, a mobile UAV relay station featuring a robotically-positioned landing platform^22^ employs external visual tracking to compensate for attitude deviations, facilitating stable landings under wind conditions reaching Beaufort scale 5.

Monocular vision systems have gained popularity in visual servoing for autonomous UAV landing owing to their cost-effectiveness and practical deployment advantages. However, when cameras are rigidly mounted on the UAV, their field of view becomes limited and often suffers from interference due to UAV motion and attitude variations. To mitigate this issue, researchers have turned to gimbal-mounted camera mechanisms. For instance, Chen et al.^23^ used GNSS to determine relative positioning, adjusting the gimbal angle, flight direction, and velocity, and implemented a Gimbal-Based Visual Servoing (GBVS) strategy to guide and execute landings. Wang et al.^24^ proposed a cooperative target-tracking framework combining gimbal cameras with multi-robot systems, leveraging an expanded field of view along with distributed Kalman filtering and swarm control to achieve collaborative mobile target tracking. Springer et al.^25^ developed a high-precision landing system employing a gimbal camera integrated with multi-sensor fusion. By utilizing wide-angle, zoom, and infrared imaging capabilities alongside visual AprilTag markers, their approach facilitated accurate landing under both daytime and nighttime conditions. Wang et al.^26^ introduced a nested landing target and detection algorithm paired with a monocular gimbal camera, enhancing both field of view and recognition reliability. Yoo et al.^27^ achieved integrated dynamic control of a multirotor UAV and gimbal camera via feedback linearization, dividing the landing sequence into horizontal and vertical phases and controlling motion using image coordinates of a single feature point, depth data, and UAV altitude. Cabecinhas et al.^28^ presented an integrated visual servoing method that actively regulates the gimbal to maintain features within the field of view, incorporating image feedback, pose estimation, and angular velocity compensation to attain stable landing control for quadrotor UAVs.

In summary, autonomous landing technology for UAVs plays a critical role in enhancing flight safety, extending mission duration, and expanding operational capabilities. Achieving autonomous landing requires a comprehensive approach that considers visual servo algorithms, estimation and compensation of landing platform motion, and overall system robustness.

Motivated by the above challenges, this paper proposes a vision-constrained gimbal-servo UAV landing control algorithm that leverages the dynamic responsiveness of a three-axis gimbal camera. The main contributions of this work are as follows:

- We formulate the UAV landing task as an image-based control problem with prescribed pixel-level FOV-constraints, thereby preventing feature-point loss and ensuring persistent visual feedback during descent.
- Unlike conventional fixed-camera approaches, the proposed method explicitly incorporates the agility of a three-axis gimbal into the controller design, which effectively enlarges the observable region and enhances tracking capability under platform motion disturbances.
- A coordinated control strategy that exploits the fast dynamics of the gimbal relative to the UAV body, enabling rapid visual tracking and enhancing overall system robustness.

## Problem formulation

### UAV system equations and variables

All coordinate systems in this paper follow the North-East-Down (NED) orientation convention: the positive *X*-axis points forward, the positive *Y*-axis points rightward, and the positive *Z*-axis points downward, orthogonal to the *XOY* plane, forming a right-handed coordinate system.

Four coordinate frames are introduced: the world frame $\{W\}$, which defines global position; the body-fixed frame $\{B\}$, whose origin is located at the UAV’s geometric center; the gimbal frame $\{G\}$, which initially coincides with $\{B\}$; and the camera frame $\{C\}$, which is aligned with $\{G\}$. Without loss of generality, the centers of mass of the UAV, gimbal, and the camera’s optical center are assumed to be co-located. Based on these frame definitions, the UAV kinematics^29^ are described as follows:

1a $$\begin{aligned} &\dot{\textbf{p}}= \textbf{v} \end{aligned}$$

1b $$\begin{aligned} &\dot{\varvec{\eta }}= \textbf{W}(\varvec{\eta }) \varvec{\omega }_b \end{aligned}$$

where $\textbf{p} = [x, y, z]^T$ and $\textbf{v} = [v_x, v_y, v_z]^T$ represent position and linear velocity in the world frame $\{W\}$. The attitude vector $\varvec{\eta } = [\phi , \theta , \psi ]^T$ denotes roll, pitch, and yaw angles, while $\varvec{\omega }_b = [\omega _x, \omega _y, \omega _z]^T$ represents angular velocity in the body frame $\{B\}$. The transformation matrix $\textbf{W}(\varvec{\eta })$ is given as follows:

2 $$\begin{aligned} \textbf{W}(\varvec{\eta }) = \begin{bmatrix} 1 & \sin \phi \tan \theta & \cos \phi \tan \theta \\ 0 & \cos \phi & -\sin \phi \\ 0 & \sin \phi / \cos \theta & \cos \phi / \cos \theta \end{bmatrix}. \end{aligned}$$

**Fig. 1:**
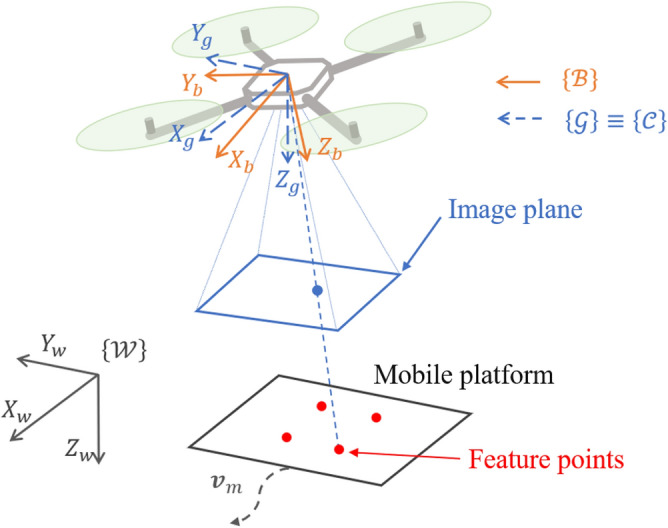
Relationship between UAV, landing platform, and coordinate frames.

The rotation matrix from $\{B\}$ to $\{W\}$, denoted $\textbf{R}_b^w \in SO(3)$, is defined as follows^30^:

3 $$\begin{aligned} \textbf{R}_b^w = \begin{bmatrix} c\theta c\psi & c\psi s\theta s\phi - s\psi c\phi & c\psi s\theta c\phi + s\psi s\phi \\ c\theta s\psi & s\psi s\theta s\phi + c\psi c\phi & s\psi s\theta c\phi - c\psi s\phi \\ -s\theta & s\phi c\theta & c\phi c\theta \end{bmatrix} \end{aligned}$$

where $c(\cdot ) = \cos (\cdot )$ and $s(\cdot ) = \sin (\cdot )$. The landing platform’s velocity and acceleration expressed in the camera frame $\{C\}$ are denoted by $\textbf{V}_m$ and $\dot{\textbf{V}}_m$, respectively. Figure 1 illustrates the spatial relationship between the UAV, the landing platform, and their associated coordinate frames.

### Gimbal-camera system description

**Fig. 2:**
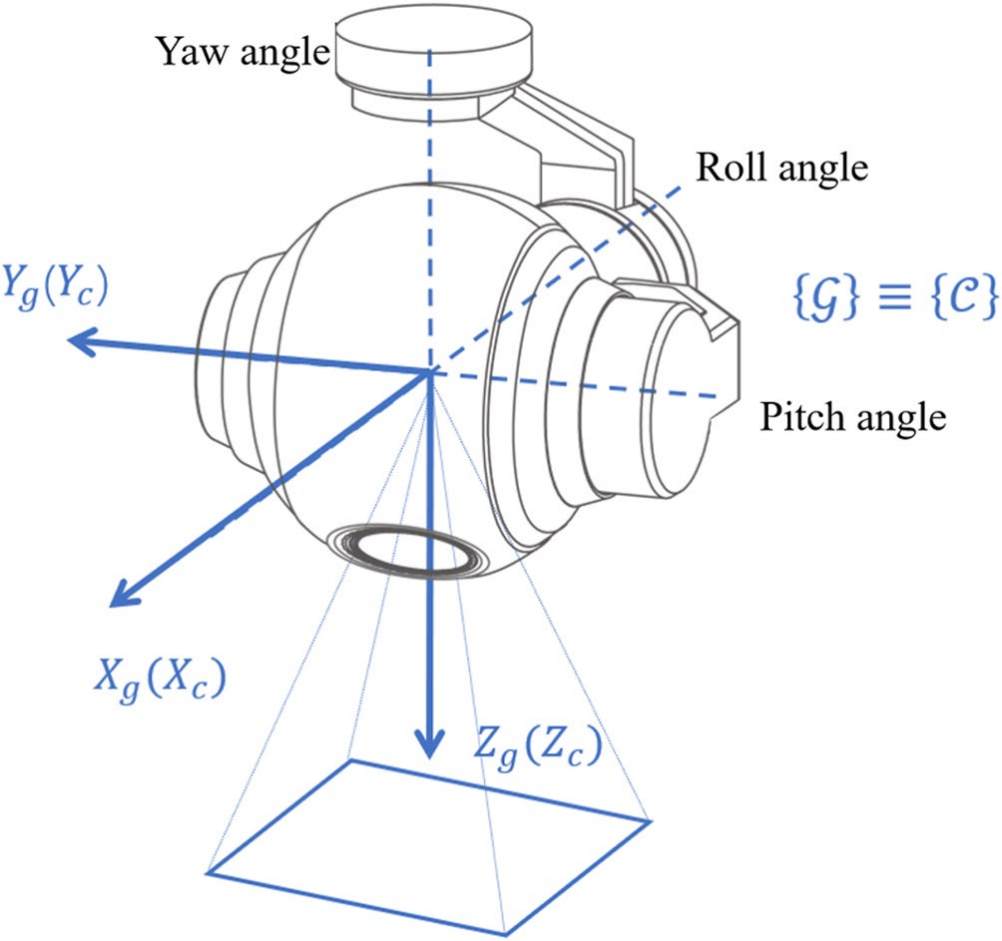
Three-axis gimbal-camera structure and associated coordinate frames.

The three-axis gimbal configuration used in this study, illustrated in Fig. 2, consists of three rotational axes corresponding to yaw (outer frame), roll (middle frame), and pitch (inner frame). The gimbal orientation is described by the angle vector $\varvec{\eta }_g = [\phi _g, \theta _g, \psi _g]^T$, where $\phi _g$, $\theta _g$, and $\psi _g$ represent the roll, pitch, and yaw angles, respectively. These angular displacements are defined relative to the UAV body frame $\{B\}$ rather than the world frame $\{W\}$.

The individual rotation matrices associated with the gimbal axes are defined as follows^31^:

4 $$\begin{aligned} \textbf{R}_{\text {out}}(\psi _g)&= \begin{bmatrix} \cos \psi _g & -\sin \psi _g & 0 \\ \sin \psi _g & \cos \psi _g & 0 \\ 0 & 0 & 1 \end{bmatrix}, \end{aligned}$$

5 $$\begin{aligned} \textbf{R}_{\text {mid}}(\phi _g)&= \begin{bmatrix} 1 & 0 & 0 \\ 0 & \cos \phi _g & -\sin \phi _g \\ 0 & \sin \phi _g & \cos \phi _g \end{bmatrix}, \quad \end{aligned}$$

6 $$\begin{aligned} \textbf{R}_{\text {in}}(\theta _g)&= \begin{bmatrix} \cos \theta _g & 0 & \sin \theta _g \\ 0 & 1 & 0 \\ -\sin \theta _g & 0 & \cos \theta _g \end{bmatrix}. \quad \end{aligned}$$

The camera coordinate frame $\{C\}$ is assumed to coincide with the gimbal frame $\{G\}$, while the gimbal base is aligned with the UAV body frame $\{B\}$. Consequently, the composite rotation matrix transforming a vector from frame $\{C\}$ to $\{B\}$ is given as follows:

7 $$\begin{aligned} \textbf{R}_c^b = \textbf{R}_g^b = \textbf{R}_{\text {out}}(\psi _g) \textbf{R}_{\text {mid}}(\phi _g) \textbf{R}_{\text {in}}(\theta _g). \end{aligned}$$

This transformation employs the ZYX Euler angle convention, where rotations occur sequentially about the yaw (*Z*-axis), pitch (*Y*-axis), and roll (*X*-axis).

### Visual dynamics analysis

In UAV landing control, analyzing the visual dynamics of target features is a key step in designing a visual servoing controller^32^. By observing the temporal variation of image features on the pixel plane, information directly related to the UAV’s motion can be extracted and utilized for control design. This subsection presents the pixel coordinate representation of feature points, analyzes their dynamic behavior, and defines the image feature error, thereby providing the theoretical foundation for subsequent controller development.

Let $P_{ci} = [x_i, y_i, z_i]^T$ denote the 3D coordinates of the $i$-th feature point expressed in the camera frame $\{C\}$, where $i = 1, 2, \ldots , n$, and $n \ge 4$. The corresponding pixel coordinates are defined as follows:

8 $$\begin{aligned} s_i = \begin{bmatrix} u_i \\ v_i \end{bmatrix} = \frac{\lambda }{z_i} \begin{bmatrix} x_i \\ y_i \end{bmatrix} \end{aligned}$$

where $\lambda$ denotes the focal length of the camera in pixels. The pixel coordinates $s_i$ are selected as the visual features, and the overall feature vector is constructed as $s = [s_1^T, \ldots , s_n^T]^T \in \mathbb {R}^{2n}$.

For a stationary target in the 3D workspace, the velocity of a feature point in $\{C\}$ is related to the spatial velocity of the camera as follows:

9 $$\begin{aligned} \dot{P}_{ci} = -\textbf{v}_c - \varvec{\omega }_c \times P_{ci} \end{aligned}$$

where $\textbf{v}_c = [v_{cx}, v_{cy}, v_{cz}]^T$ and $\varvec{\omega }_c = [\omega _{cx}, \omega _{cy}, \omega _{cz}]^T$ denote the translational and angular velocities of the camera, respectively, expressed in $\{C\}$. Expanding equation (9) yields as follows:

10 $$\begin{aligned} {\left\{ \begin{array}{ll} \dot{x}_i = -v_{cx} - \omega _{cy} z_i + \omega _{cz} y_i \\ \dot{y}_i = -v_{cy} - \omega _{cz} x_i + \omega _{cx} z_i \\ \dot{z}_i = -v_{cz} - \omega _{cx} y_i + \omega _{cy} x_i. \end{array}\right. } \end{aligned}$$

Differentiating the image feature expression (8) with respect to time, the pixel dynamics are obtained as follows:

11 $$\begin{aligned} {\left\{ \begin{array}{ll} \dot{u}_i = \frac{\lambda }{z_i} \dot{x}_i - \frac{\lambda x_i}{z_i^2} \dot{z}_i \\ \dot{v}_i = \frac{\lambda }{z_i} \dot{y}_i - \frac{\lambda y_i}{z_i^2} \dot{z}_i. \end{array}\right. } \end{aligned}$$

Substituting equation (10) into (11) yields the time derivative of the pixel coordinates as follows:

12 $$\begin{aligned} {\left\{ \begin{array}{ll} \dot{u}_i = -\frac{\lambda }{x_i} v_{cx} + \frac{u_i}{x_i} v_{cz} + \frac{u_i v_i}{\lambda } \omega _{cx} - \frac{\lambda ^2 + u_i^2}{\lambda } \omega _{cy} + v_i \omega _{cz} \\ \dot{v}_i = -\frac{\lambda }{x_i} v_{cy} + \frac{v_i}{x_i} v_{cz} + \frac{\lambda ^2 + v_i^2}{\lambda } \omega _{cx} - \frac{u_i v_i}{\lambda } \omega _{cy} - u_i \omega _{cz}. \end{array}\right. } \end{aligned}$$

Rearranging equation (12), the image feature dynamics can be compactly expressed as follows:

13 $$\begin{aligned} \dot{s}_i = L_i(z_i, s_i) \varvec{V}_c \end{aligned}$$

where $\varvec{V}_c \triangleq [\textbf{v}_c^T, \varvec{\omega }_c^T]^T \in \mathbb {R}^6$ is the camera velocity vector in the camera frame $\{C\}$, consisting of translational and angular components. $L_i(z_i, s_i)$ denotes the image Jacobian matrix associated with the *i*-th feature point and is given as follows:

14 $$\begin{aligned} L_i(z_i, s_i) = \begin{bmatrix} -\frac{\lambda }{z_i} & 0 & \frac{u_i}{z_i} & \frac{u_i v_i}{\lambda } & -\frac{\lambda ^2 + u_i^2}{\lambda } & v_i \\ 0 & -\frac{\lambda }{z_i} & \frac{v_i}{z_i} & \frac{\lambda ^2 + v_i^2}{\lambda } & -\frac{u_i v_i}{\lambda } & -u_i \end{bmatrix}. \end{aligned}$$

In this matrix, $z_i$ represents the depth of the *i*-th feature point in the camera frame. Given the physical constraints of the UAV airframe and the camera’s focal length, the depth $z_i$ of the *i*-th feature point in the camera frame is bounded below by a positive minimum value, which prevents singularities in (11). Considering a total of *n* feature points in the visual servoing process, the overall image Jacobian matrix is defined as $L(z, s) = [L_1^T, \ldots , L_n^T]^T \in \mathbb {R}^{2n \times 6}$, and the system dynamics can be written as follows:

15 $$\begin{aligned} \dot{s} = L(z, s) \varvec{V}_c \end{aligned}$$

where $z = [z_1, \ldots , z_n]^T \in \mathbb {R}^n$ is the feature depth vector.

For a moving landing platform undergoing pure translation with velocity $\textbf{v}_m$ in the camera frame $\{C\}$, the motion of its feature points is likewise $\textbf{v}_m$. Accordingly, the full velocity vector of the platform is defined as $\varvec{V}_m = [\textbf{v}_m^T, 0, 0, 0]^T$. Considering the relative motion between the camera and the platform, we reformulate equations (9) to (15) as follows:

16 $$\begin{aligned} \dot{s} = L(z, s)(\varvec{V}_c - \varvec{V}_m). \end{aligned}$$

This equation characterizes the image feature dynamics under relative motion between the UAV and the moving platform.

The image feature tracking error is defined as follows:

17 $$\begin{aligned} e_s = s - s_d \end{aligned}$$

where $e_s = [e_{s1}^T, \ldots , e_{sn}^T]^T = [e_{u1}, e_{v1}, \ldots , e_{un}, e_{vn}]^T \in \mathbb {R}^{2n}$ denotes the pixel coordinate error of the feature points, and $s_d = [s_{d1}^T, \ldots , s_{dn}^T]^T \in \mathbb {R}^{2n}$ represents the desired pixel coordinates corresponding to the UAV’s ideal landing position. These desired features are predefined and time-invariant, i.e., $\dot{s}_d = 0$. Therefore, the time derivative of the image feature error is as follows:

18 $$\begin{aligned} \dot{e}_s = \dot{s} - \dot{s}_d = L(z, s)(\varvec{V}_c - \varvec{V}_m). \end{aligned}$$

### Field-of-view constraints

During the UAV landing process, constraints can be imposed on the feature pixel coordinates to ensure that image features remain within the camera’s FOV^33^. The upper and lower bounds of the feature point pixel coordinates are defined as follows:

19a $$\begin{aligned} u_{\text {min}}&\le u_i \le u_{\text {max}} \end{aligned}$$

19b $$\begin{aligned} v_{\text {min}}&\le v_i \le v_{\text {max}} \end{aligned}$$

where $u_{\text {min}}, u_{\text {max}}, v_{\text {min}}, v_{\text {max}}$ denote the lower and upper bounds of the camera’s pixel coordinates or designer-preset pixel coordinate ranges.

**Fig. 3:**
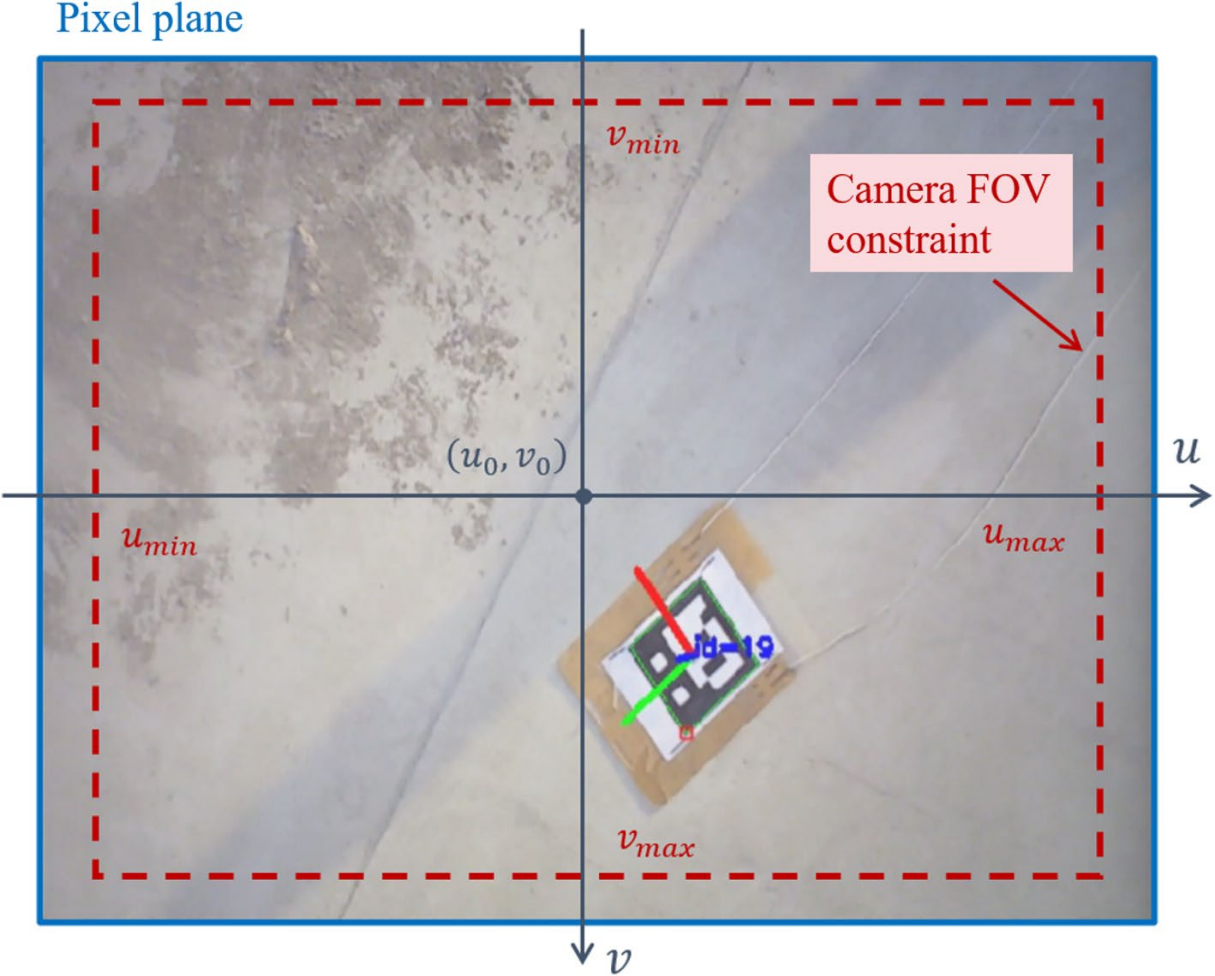
Gimbal attitude angles during landing maneuver.

As illustrated in Fig. 3, the upper and lower bounds of the feature point pixel coordinates constrain the position of feature points within the camera’s FOV.

Let $\underline{e}_{ui,0}, \overline{e}_{ui,0}, \underline{e}_{vi,0}, \overline{e}_{vi,0}$ represent the initial upper and lower bounds of the feature point pixel coordinate errors as follows:

20a $$\begin{aligned} \underline{e}_{ui,0} = u_{di} - u_{\text {min}}, \quad \overline{e}_{ui,0} = u_{\text {max}} - u_{di} \end{aligned}$$

20b $$\begin{aligned} \underline{e}_{vi,0} = v_{di} - v_{\text {min}}, \quad \overline{e}_{vi,0} = v_{\text {max}} - v_{di} . \end{aligned}$$

To ensure prescribed transient and steady-state performance of image feature errors, the image feature error $e_{si}$ must satisfy constraint conditions described by the inequalities as follows:

21a $$\begin{aligned} -\underline{e}_{ui,0}\rho _{ui}(t) \le e_{ui}(t) \le \overline{e}_{ui,0}\rho _{ui}(t) \end{aligned}$$

21b $$\begin{aligned} -\underline{e}_{vi,0}\rho _{vi}(t) \le e_{vi}(t) \le \overline{e}_{vi,0}\rho _{vi}(t) \end{aligned}$$

where $\rho _{ui}(t), \rho _{vi}(t)$ are performance functions for the feature point pixel coordinate errors, specifying the time-varying upper and lower bounds for error evolution during transient and steady states. They are defined as follows:

22a $$\begin{aligned} \rho _{ui}(t) = \left( 1 - \frac{e_{ui,\infty }}{c_{ui}}\right) \exp \left( -\kappa _{ui}t\right) + \frac{e_{ui,\infty }}{c_{ui}} \end{aligned}$$

22b $$\begin{aligned} \rho _{vi}(t) = \left( 1 - \frac{e_{vi,\infty }}{c_{vi}}\right) \exp \left( -\kappa _{vi}t\right) + \frac{e_{vi,\infty }}{c_{vi}} \end{aligned}$$

where $c_{ui} = \max \{\underline{e}_{ui,0}, \overline{e}_{ui,0}\}$ and $c_{vi} = \max \{\underline{e}_{vi,0}, \overline{e}_{vi,0}\}$. Constants $e_{ui,\infty }, e_{vi,\infty } > 0$ represent the steady-state performance indices for the feature point pixel coordinate errors, and $\kappa _{ui}, \kappa _{vi} > 0$ are the decay rates of the performance functions. For $e_{ui,\infty }, e_{vi,\infty }$, setting $e_{ui,\infty } < c_{ui}$ and $e_{vi,\infty } < c_{vi}$ ensures effective convergence of the errors at the prescribed rate.

### Control objective

This work builds upon the following reasonable assumptions and effective lemmas for the design of the control strategy. All assumptions hold within the valid scope of the physical world and can be stably achieved in practical applications through preliminary positioning control of the camera and UAV.

**Assumption 1:** The velocity and acceleration vectors of the landing platform, $\varvec{V}_m, \dot{\varvec{V}}_m \in \mathbb {R}^6$, are bounded but unknown^34^, satisfying as follows:

23 $$\begin{aligned} \Vert \varvec{V}_m\Vert< \overline{v}_{m1}, \quad \Vert \dot{\varvec{V}}_m\Vert < \overline{v}_{m2}. \end{aligned}$$

**Assumption 2:** At the initiation of the landing procedure, all image feature points reside within the camera’s FOV.

Under Assumption 2, the initial pixel coordinate errors of the image features are bounded by the FOV-constraints. Consequently, the initial conditions for equation () are given as follows:

24a $$\begin{aligned} -e_{ut,0}&\le e_{ut}(0) \le \overline{e}_{ut,0} \end{aligned}$$

24b $$\begin{aligned} -e_{vi,0}&\le e_{vi}(0) \le \overline{e}_{vi,0}. \end{aligned}$$

Thus, the FOV-constraints problem () is transformed into regulating the transient and steady-state performance of the feature point pixel coordinate errors, as specified in equation ().

**Control Objective:** Given Assumptions 1 and 2, and considering a moving platform with unknown velocity $\varvec{V}_m$, design a three-axis gimbal UAV visual servo controller $\varvec{V}_c$ for the UAV position and attitude system model. The controller must ensure that:

1. The feature points ultimately converge to a bounded neighborhood of the desired pixel coordinates $\textbf{s}^*$, enabling successful UAV landing.
2. The image feature error (17) satisfies the performance constraint () for all time $t \ge 0$.

## Controller design

**Fig. 4:**
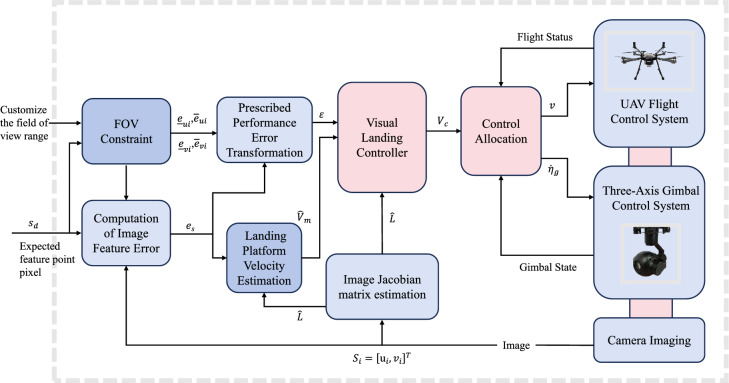
Block diagram of vision-constrained gimbal-servo UAV landing control system.

This section presents the design of a vision-based servo controller to enable the precise landing of a UAV equipped with a gimbal-mounted camera. The proposed controller ensures that the pixel coordinates of image feature points converge to a bounded neighborhood of their desired values, while strictly satisfying the FOV-constraints defined in equation (21). The system architecture is illustrated in Fig. 4.

### Error transformation

To enforce the FOV-constraints and guarantee both steady-state and transient performance, the image feature error $e_s$ is mapped into an unconstrained space through a smooth and strictly increasing transformation function. The normalized error is defined as follows:

25a $$\begin{aligned} \xi _{ui}(t)&= \frac{e_{ui}}{\rho _{ui}} \end{aligned}$$

25b $$\begin{aligned} \xi _{vi}(t)&= \frac{e_{vi}}{\rho _{vi}} \end{aligned}$$

for $i = 1, \ldots , n$, where $\rho _{ui}(t)$ and $\rho _{vi}(t)$ are performance functions specified in equation (22). The transformed error is then defined as follows:

26a $$\begin{aligned} \varepsilon _{ui}(t)&= k_{ui} \ln \left( \frac{\underline{e}_{ui,0} + \xi _{ui}}{\overline{e}_{ui,0} - \xi _{ui}} \right) \end{aligned}$$

26b $$\begin{aligned} \varepsilon _{vi}(t)&= k_{vi} \ln \left( \frac{\underline{e}_{vi,0} + \xi _{vi}}{\overline{e}_{vi,0} - \xi _{vi}} \right) \end{aligned}$$

where $k_{ui} = \frac{\overline{e}_{ui,0} \cdot \underline{e}_{ui,0}}{\overline{e}_{ui,0} + \underline{e}_{ui,0}}$ and $k_{vi} = \frac{\overline{e}_{vi,0} \cdot \underline{e}_{vi,0}}{\overline{e}_{vi,0} + \underline{e}_{vi,0}}$ are constants derived from the initial error bounds given in equation (). The overall transformed error vector is given as follows:

27 $$\begin{aligned} \varepsilon (s,t) = [\varepsilon _{u1}, \varepsilon _{v1}, \ldots , \varepsilon _{un}, \varepsilon _{vn}]^T. \end{aligned}$$

### Vision-based servo controller

The vision-based servo controller generates velocity commands for the camera frame $\{C\}$ to regulate the image feature point errors toward zero as follows:

28 $$\begin{aligned} \varvec{V}_c= -k \hat{L}^+ \varepsilon (s,t) + \varvec{\hat{V}}_m \end{aligned}$$

where $k > 0$ is a controller gain, $\hat{V}_m$ is the estimated velocity of the landing platform, and $\hat{L}^+$ is the pseudoinverse of the estimated image Jacobian $\hat{L}$. The pseudoinverse $\hat{L}^+$ is computed as follows:

29 $$\begin{aligned} \hat{L}^+ \triangleq \left( \hat{L}^T \hat{L} \right) ^{-1} \hat{L}^T. \end{aligned}$$

If $\hat{L}$ has full column rank, then $\hat{L}^+ \hat{L} = I$.

Substituting (28) into the error dynamics in equation (18) yields as follows:

30 $$\begin{aligned} \dot{e}_s = -k L \hat{L}^+ \varepsilon - L e_m \end{aligned}$$

where

31 $$\begin{aligned} e_m = \varvec{V}_m - \varvec{\hat{V}}_m \end{aligned}$$

is the estimation error of the landing platform’s velocity.

### Landing platform velocity estimation

To estimate the unknown landing platform velocity $\varvec{V}_m$, an observer is designed based on the measurable camera velocity $\varvec{V}_c$ and image feature error $e_s$. Low-pass filters are employed to extract temporal derivatives as follows^35^:

32 $$\begin{aligned} \mu _1 \dot{e}_{fs} + e_{fs}&= e_s, \quad e_{fs}(0) = 0 \end{aligned}$$

33 $$\begin{aligned} \mu _1 \dot{h} + h&= \hat{L} \varvec{V}_c, \quad h(0) = 0 \end{aligned}$$

34 $$\begin{aligned} \mu _1 \dot{g} + g&= e_s, \quad g(0) = 0 \end{aligned}$$

where $\mu _1 > 0$ is a time constant, and $e_{fs}(t), h(t), g(t) \in \mathbb {R}^{2n}$ are the filter states. Solving Eq. 34) yields as follows:

35 $$\begin{aligned} g(t) = \frac{1}{\mu _1} \left( e_s(t) - e_{fs}(t) - \exp \left( -\frac{1}{\mu _1}t\right) e_s(0) \right) . \end{aligned}$$

The filtered signal $S(t) \in \mathbb {R}^{2n}$, representing $\hat{L} \varvec{V}_m$, satisfies the dynamics as follows:

36 $$\begin{aligned} \dot{S} = \frac{1}{\mu _1} \left( \hat{L} \varvec{V}_m - S \right) , \quad S(0) = 0 \end{aligned}$$

with the identity $g(t) = h(t) - S(t)$.

Thus, the estimate of $\hat{L} \varvec{V}_m$ is as follows:

37 $$\begin{aligned} S = h - g \end{aligned}$$

and the estimated platform velocity is as follows:

38 $$\begin{aligned} \hat{\varvec{V}}_m = \hat{L}^+ S. \end{aligned}$$

To mitigate the peaking phenomenon caused by an improper choice of $\mu _1$, a projection-based differential estimator is adopted as follows:

39 $$\begin{aligned} \begin{aligned} \dot{\hat{\varvec{V}}}_m&= \text {Proj}(\varvec{\hat{V}}_m, \phi _m) = {\left\{ \begin{array}{ll} \phi _m - \varphi _m,&\begin{array}{c} \text {if } f(\varvec{\hat{V}}_m)> 0 \text { and } \phi _m^T \nabla f(\varvec{\hat{V}}_m) > 0 \end{array} \\ \phi _m, & \text {otherwise} \end{array}\right. } \end{aligned} \end{aligned}$$

with the initial condition $\varvec{\hat{V}}_m(0) = 0$. Here, $\varphi _m$ is defined as follows:

40 $$\begin{aligned} \varphi _m \triangleq \frac{\nabla f(\varvec{\hat{V}}_m)\nabla f(\varvec{\hat{V}}_m)^T}{\Vert \nabla f(\varvec{\hat{V}}_m)\Vert ^2} \phi _m f(\varvec{\hat{V}}_m) \end{aligned}$$

which represents the weighted projection of the estimation error $\phi _m \triangleq \frac{1}{\mu _2} (\hat{L}^+ S - \varvec{\hat{V}}_m)$, where $\mu _2 > 0$ is a design parameter. The error function and its gradient are given as follows:

41 $$\begin{aligned} f(\varvec{\hat{V}}_m)&= \frac{\Vert \varvec{\hat{V}}_m\Vert ^2 - h_1^2}{\varepsilon _0 h_1^2} \end{aligned}$$

42 $$\begin{aligned} \nabla f(\varvec{\hat{V}}_m)&= \frac{2\varvec{\hat{V}}_m}{\varepsilon _0 h_1^2} \end{aligned}$$

where $h_1 > 0$ denotes the upper bound on the velocity magnitude of $\varvec{V}_m$, and $\varepsilon _0 > 0$ specifies the tolerance in velocity estimation. The projection law in equation (39) is then employed to ensure boundedness of the estimate $\varvec{\hat{V}}_m$.

### Control allocation

According to equation (28), the designed visual servoing controller $\varvec{V}_c$ must be decomposed into executable kinematic commands for both the UAV and the three-axis gimbal. Specifically, the control inputs consist of the UAV translational velocity $\textbf{v}$ and the gimbal angular velocity $\dot{\varvec{\eta }}_g = [\dot{\phi }_g, \dot{\theta }_g, \dot{\psi }_g]^\textrm{T}$.

First, by combining equations (3) and (7), the transformation from the desired camera translational velocity $v_c$ to the UAV velocity $\textbf{v}$ in the world coordinate frame $\{Vv\}$ is derived as follows:

43 $$\begin{aligned} \begin{aligned} \textbf{v}&= R_v^w R_c^b v_c = R_v^w R_{\text {out}}(\psi _g) R_{\text {mid}}(\phi _g) R_{\text {in}}(\theta _g) v_c. \end{aligned} \end{aligned}$$

Next, based on the 3D geometric relationship between the UAV and the gimbal (as shown in equation (7) and Fig. 2), the camera angular velocity $\omega _c$ can be expressed as follows:

44 $$\begin{aligned} \begin{aligned} \omega _c&= R_v^c \omega _b + R_{\text {in}}^\textrm{T}(\theta _g) R_{\text {mid}}(\phi _g) \dot{\psi }_g + R_{\text {in}}^\textrm{T}(\theta _g) \dot{\phi }_g + \dot{\theta }_g \\&= R_{\text {in}}^\textrm{T}(\theta _g) R_{\text {mid}}(\phi _g) R_{\text {out}}(\psi _g) \omega _b + R_{\text {in}}^\textrm{T}(\theta _g) R_{\text {mid}}(\phi _g) \dot{\psi }_g + R_{\text {in}}^\textrm{T}(\theta _g) \dot{\phi }_g + \dot{\theta }_g \end{aligned} \end{aligned}$$

where $\dot{\psi }_g = [0, 0, \dot{\psi }_g]^\textrm{T}$, $\dot{\phi }_g = [\dot{\phi }_g, 0, 0]^\textrm{T}$, and $\dot{\theta }_g = [0, \dot{\theta }_g, 0]^\textrm{T}$ represent the vector forms of the gimbal angular velocities.

Given the current gimbal orientation $\varvec{\eta }_g = [\phi _g, \theta _g, \psi _g]^\textrm{T}$ and the UAV angular velocity $\varvec{\omega }_b = [\omega _x, \omega _y, \omega _z]^\textrm{T}$, the gimbal angular velocity can be calculated as follows:

45 $$\begin{aligned} \dot{\varvec{\eta }}_g = M \left( \omega _c - R_{\text {in}}^\textrm{T}(\theta _g) R_{\text {mid}}^\textrm{T}(\phi _g) R_{\text {out}}^\textrm{T}(\psi _g) \varvec{\omega }_b \right) \end{aligned}$$

where the transformation matrix *M* is defined as follows:

46 $$\begin{aligned} M = \begin{bmatrix} \cos \theta _g & 0 & \sin \theta _g \\ \sin \theta _g \tan \phi _g & 1 & -\cos \theta _g \tan \phi _g \\ -\sin \theta _g / \cos \phi _g & 0 & \cos \theta _g / \cos \phi _g \end{bmatrix}. \end{aligned}$$

Note that when $\phi _g = \pm \pi /2$, the matrix *M* becomes singular. Therefore, in practical applications, the gimbal pitch angle $\phi _g$ must be constrained to satisfy $-\pi /2< \phi _g < \pi /2$.

In practice, control allocation is achieved by applying equations (43) and (45) to compute the desired UAV translational velocity $\textbf{v}$ and the gimbal angular velocity $\dot{\varvec{\eta }}_g$. These control commands are then sent to the UAV flight controller and the gimbal controller, respectively, to execute the landing maneuver.

### Stability analysis

Under Assumptions 1 and 2, for the UAV kinematic model (1), visual dynamics (16), control law (28), and velocity estimator (73), there exist sufficiently large $k > 0$ and sufficiently small $\mu _1, \mu _2 > 0$ such that:

1. The image feature error $e_s$, velocity estimation error $e_m$, and associated filter states converge to an arbitrarily small neighborhood of the origin. The size of this neighborhood can be tuned via design parameters.
2. Both transient and steady-state performances of $e_s$ are guaranteed while strictly satisfying the FOV-constraints given in (21).
3. All signals in the closed-loop system remain uniformly ultimately bounded (UUB).

**Step 1: Existence and Uniqueness:** Define the normalized error vector $\varvec{\xi } \triangleq [\xi _{u1}, \xi _{v1}, \ldots , \xi _{un}, \xi _{vn}]^\textrm{T}$ and the open set:

$$\begin{aligned} \Omega _\xi = \biggl \{&(\xi , t) \in \mathbb {R}^{2n} \times \mathbb {R}_{\ge 0} \biggm | \xi \in \prod _{i=1}^n \left( -\underline{\epsilon }_{ui,0}, \overline{\epsilon }_{ui,0} \right) \times \left( -\underline{\epsilon }_{vi,0}, \overline{\epsilon }_{vi,0} \right) \biggr \}. \end{aligned}$$

Under Assumption 2 and the continuity of the closed-loop system dynamics, a unique solution $\varvec{\xi }(t) \in \Omega _\xi$ exists for $t \in [0, \tau _{\max })$.

**Step 2: Boundedness and Convergence:** We now show that the proposed controller (28) ensures the validity of stability analysis and that the results remain valid as $\tau _{\max } \rightarrow \infty$.

Let the filter error for estimating the visual target’s velocity be defined as follows:

47 $$\begin{aligned} \dot{\varvec{e}}_f = -\frac{1}{\mu _1} \varvec{e}_f + \dot{\hat{\varvec{L}}} \varvec{V}_m + \hat{\varvec{L}} \dot{\varvec{V}}_m \end{aligned}$$

where $$\varvec{e}_f = \hat{\varvec{L}} \varvec{V}_m - \varvec{S}$$.

Given the velocity estimation error $\varvec{e}_m = \varvec{V}_m - \hat{\varvec{V}}_m$ and the projection-based estimator (39), we have as follows:

48 $$\begin{aligned} \begin{aligned} \dot{\varvec{e}}_m&= \dot{\varvec{V}}_m - \frac{1}{\mu _2} \left( \hat{\varvec{L}}^+ \varvec{S} + \varvec{e}_m - \varvec{V}_m \right) + \varvec{\varphi }_m. \end{aligned} \end{aligned}$$

Assuming $\hat{\varvec{L}}$ is full column rank, which holds in practice due to the positive depth $z_i$ and focal length $\lambda$, we have $\hat{\varvec{L}}^+ \hat{\varvec{L}} = I$. Using this, equation (48) becomes as follows:

49 $$\begin{aligned} \begin{aligned} \dot{\varvec{e}}_m&= -\frac{1}{\mu _2} \varvec{e}_m + \frac{1}{\mu _2} \hat{\varvec{L}}^+ \varvec{e}_f + \dot{\varvec{V}}_m + \varvec{\varphi }_m. \end{aligned} \end{aligned}$$

From the performance function (22) and transformed error dynamics in equations (26)-(27), the derivative of the transformed error $\varvec{\varepsilon }$ is as follows:

50 $$\begin{aligned} \begin{aligned} \dot{\varvec{\varepsilon }}&= \frac{\partial \varvec{\varepsilon }}{\partial \varvec{\xi }} \left( \frac{\partial \varvec{\xi }}{\partial \varvec{e}_s} \dot{\varvec{e}}_s + \frac{\partial \varvec{\xi }}{\partial \varvec{\rho }} \dot{\varvec{\rho }} \right) \\&= \frac{\partial \varvec{\varepsilon }}{\partial \varvec{\xi }} \left( \frac{\partial \varvec{\xi }}{\partial \varvec{e}_s} \varvec{L}(\varvec{V}_c - \varvec{V}_m) + \frac{\partial \varvec{\xi }}{\partial \varvec{\rho }} \dot{\varvec{\rho }} \right) \\&= -k \frac{\partial \varvec{\varepsilon }}{\partial \varvec{\xi }} \frac{\partial \varvec{\xi }}{\partial \varvec{e}_s} \varvec{L} \hat{\varvec{L}}^+ \varvec{\varepsilon } - \frac{\partial \varvec{\varepsilon }}{\partial \varvec{\xi }} \frac{\partial \varvec{\xi }}{\partial \varvec{e}_s} \varvec{L} \varvec{e}_m + \frac{\partial \varvec{\varepsilon }}{\partial \varvec{\xi }} \frac{\partial \varvec{\xi }}{\partial \varvec{\rho }} \dot{\varvec{\rho }}. \end{aligned} \end{aligned}$$

Define the task variable $\varvec{\varepsilon }_L \triangleq \hat{\varvec{L}}^+ \varvec{\varepsilon }$, and for a constant $r > 0$, define the set:

$$\Omega _{\varepsilon } \triangleq \left\{ (\varvec{\xi }, t) \in \mathbb {R}^{2n} \times [0, \tau _{\max }) \mid \Vert \varvec{\varepsilon }_L\Vert < r \right\} \subset \Omega _\xi .$$

Then, the time derivative of $\varvec{\varepsilon }_L$ is given as follows:

$$\begin{aligned} \begin{aligned} \dot{\varvec{\varepsilon }}_L&= \frac{d \hat{\varvec{L}}^+}{dt} \varvec{\varepsilon } + \hat{\varvec{L}}^+ \dot{\varvec{\varepsilon }}= \varvec{O}(\varvec{e}_s,t)(\varvec{V}_c - \varvec{V}_m) + \hat{\varvec{L}}^+ \dot{\varvec{\varepsilon }}. \end{aligned} \end{aligned}$$

According to reference^36^, $\frac{d \hat{\varvec{L}}^+}{dt} \varvec{\varepsilon } = \varvec{O}(\varvec{e}_s,t)(\varvec{V}_c - \varvec{V}_m)$, where $\varvec{O}(\varvec{e}_s,t)$ is a matrix satisfying $\varvec{O}({\varvec{e}}_s, t)\big |_{{\varvec{e}}_s=0} = 0_{6 \times 6}$.

Substituting the controller (28) and velocity estimation error (31), we obtain as follows:

51 $$\begin{aligned} \begin{aligned} \dot{\varvec{\varepsilon }}_L&= (\varvec{O}(\varvec{e}_s,t) + \varvec{m}(t))(-k \varvec{\varepsilon }_L - \varvec{e}_m) + \varvec{n}(t) \end{aligned} \end{aligned}$$

where

$$m(t) = \hat{\varvec{L}}^+ \frac{\partial \varvec{\varepsilon }}{\partial \varvec{\xi }} \frac{\partial \varvec{\xi }}{\partial \varvec{e}_s} \varvec{L}, \quad n(t) = \hat{\varvec{L}}^+ \frac{\partial \varvec{\varepsilon }}{\partial \varvec{\xi }} \frac{\partial \varvec{\xi }}{\partial \varvec{\rho }} \dot{\varvec{\rho }} .$$

Linearizing equation (51) around $\varvec{\varepsilon }_L = 0$ yields as follows:

52 $$\begin{aligned} \dot{\varvec{\varepsilon }}_L = \varvec{A}(t) \varvec{\varepsilon }_L + \varvec{B}(t) \varvec{e}_m + \varvec{C}(t) \end{aligned}$$

where $\varvec{A}(t)$, $\varvec{B}(t)$, and $\varvec{C}(t)$ are bounded time-varying matrices depending on *m*(*t*), $\varvec{O}(t)$, and $\varvec{n}(t)$. where $\varvec{A}(t)$, $\varvec{B}(t)$, and $\varvec{C}(t)$ are defined as follows:

53 $$\begin{aligned} \begin{aligned} \varvec{A}(t)&= -k \varvec{m}(t) \bigg |_{\varepsilon _L=0} \\ \varvec{B}(t)&= \frac{\partial }{\partial \varepsilon _L} \left( \varvec{n}(t) - \varvec{m}(t) e_m \right) \bigg |_{\varepsilon _L=0} \\ \varvec{C}(t)&= \left( \varvec{n}(t) - \varvec{m}(t) e_m\right) \bigg |_{\varepsilon _L=0}. \end{aligned} \end{aligned}$$

When feature points remain within the image FOV and both true depth values and estimates $\textbf{e}_i$ are positive, the boundedness and convergence of $\varvec{n}(t)$ follow directly from the convergence properties of the performance function (21) for $t \in [0, \tau _{\text {max}})$. Furthermore, since $\frac{\partial \varepsilon }{\partial \xi } \frac{\partial \xi }{\partial e_s}$ in $\varvec{m}(t)$ constitutes a diagonal positive definite matrix, $\varvec{m}(t)$ is bounded and Lipschitz continuous. By Theorems 4.12 and 4.13 in^37^ , for sufficiently large control gain $k$, there exist constants $c_1, c_2 > 0$ such that a continuously differentiable, bounded, positive definite diagonal matrix $P(t)$ satisfies as follows:

54 $$\begin{aligned} 0 < c_1 I \le \varvec{P}(t) \le c_2 I \end{aligned}$$

and the differential equation as follows:

55 $$\begin{aligned} -\dot{\varvec{P}}(t) = \varvec{P}(t)\varvec{A}(t) + \varvec{A}^T(t)\varvec{P}(t) + \varvec{Q}(t) \end{aligned}$$

where $\varvec{Q}(t)$ is a continuous positive definite diagonal matrix with $\varvec{Q}(t) \ge c_3 I > 0$ for constant $c_3 > 0$.

Given the form of $\varvec{B}(t)$, its Lipschitz continuity over $t \in [0, t_{\max })$ implies $\Vert \varvec{B}(t) \Vert \le c_4 \Vert \varvec{\varepsilon }_L \Vert \Vert \varvec{e}_m \Vert$ for Lipschitz constant $c_4 > 0$. Similarly, the boundedness of $\varvec{C}(t)$ yields $\Vert \varvec{C}(t) \Vert \le c_5 \Vert \varvec{e}_m \Vert$ with $c_5 > 0$.

Consider the Lyapunov function candidate as follows:

56 $$\begin{aligned} V = {\varvec{\varepsilon }}_{L}^{T } {\varvec{P}}(t) {\varvec{\varepsilon }}_{L} + \frac{1}{2} {\varvec{e}}_{m}^{T} {\varvec{e}}_{m} + \frac{1}{2} {\varvec{e}}_{f}^{T} {\varvec{e}}_{f}. \end{aligned}$$

Differentiating equation (56) using equations (47) and (49) gives as follows:

57 $$\begin{aligned} \begin{aligned} \dot{V}&= {\varvec{\varepsilon }}_L^T {\varvec{P}}(t) \dot{{\varvec{\varepsilon }}}_L + \dot{{\varvec{\varepsilon }}}_L^T {\varvec{P}}(t) {\varvec{\varepsilon }}_L + {\varvec{\varepsilon }}_L^T \dot{{\varvec{P}}}(t) {\varvec{\varepsilon }}_L \\&+ {\varvec{e}}_m^T \left( -\frac{1}{\mu _2}{\varvec{e}}_m + \frac{1}{\mu _2}\hat{{\varvec{L}}}^\dagger {\varvec{e}}_f + \dot{{\varvec{V}}}_m + {\varvec{\varphi }}_m \right) \\&+ {\varvec{e}}_f^T \left( -\frac{1}{\mu _1}{\varvec{e}}_f + \dot{\hat{{\varvec{L}}}} {\varvec{V}}_m + \hat{{\varvec{L}}} \dot{{\varvec{V}}}_m \right) . \end{aligned} \end{aligned}$$

Substituting equations (52) and (55) yields as follows:

58 $$\begin{aligned} \begin{aligned} \dot{V}&= -{\varvec{\varepsilon }}_L^T {\varvec{Q}}(t) {\varvec{\varepsilon }}_L + 2{\varvec{\varepsilon }}_L^T {\varvec{P}}(t) {\varvec{B}}(t) {\varvec{\varepsilon }}_L + 2{\varvec{\varepsilon }}_L^T {\varvec{P}}(t) {\varvec{C}}(t) \\&+ {\varvec{e}}_m^T \left( -\frac{1}{\mu _2}{\varvec{e}}_m + \frac{1}{\mu _2}\hat{{\varvec{L}}}^+ {\varvec{e}}_f + \dot{{\varvec{V}}}_m + {\varvec{\varphi }}_m \right) \\&+ {\varvec{e}}_f^T \left( -\frac{1}{\mu _1}{\varvec{e}}_f + \dot{\hat{{\varvec{L}}}} {\varvec{V}}_m + \hat{{\varvec{L}}} \dot{{\varvec{V}}}_m \right) . \end{aligned} \end{aligned}$$

During landing with feature points within FOV, the camera height exceeds the landing platform height, ensuring image depth $z_i > 0$. Physical constraints of the UAV frame and camera focal length establish a lower bound $\underline{z_i} = \min (h_c, f_c) > 0$, where $h_c$ is the distance from the gimbal center to the contact point and $f_c$ the focal length. By Assumption 1 and equation (), boundedness of $s_i$ at $t=0$ combined with equations (5), (14), and (17) (noting constant $s_d$) implies as follows:

59 $$\begin{aligned} \Vert \hat{{\varvec{L}}}\Vert \le \sigma _h(\Vert {\varvec{e}}_s\Vert ) \end{aligned}$$

where $\sigma _h(\cdot )$ increases monotonically with $\Vert {\varvec{e}}_s\Vert$. Similarly, equation (29) gives as follows:

60 $$\begin{aligned} \Vert \hat{{\varvec{L}}}^+\Vert \le \sigma _p(\Vert {\varvec{e}}_s\Vert ) \end{aligned}$$

for monotonic $$\sigma _p(\cdot )$$.

Define $\hat{\varvec{P}}_{ci} = [x_{1i}, x_{2i}, x_{3i}]^T = [u_i, v_i, z_i^{-1}]^T$. The image Jacobian (14) becomes as follows:

61 $$\begin{aligned} \hat{{\varvec{L}}}_i^T = \begin{bmatrix} -\lambda x_{3i} & 0 \\ 0 & -\lambda x_{3i} \\ x_{1i}x_{3i} & x_{2i}x_{3i} \\ \lambda ^{-1}x_{1i}x_{2i} & \lambda ^{-1}(\lambda ^2+x_{2i}^2) \\ -\lambda ^{-1}(\lambda ^2+x_{1i}^2) & -\lambda ^{-1}x_{1i}x_{2i} \\ x_{2i} & -x_{1i} \end{bmatrix} \end{aligned}$$

with time derivative as follows:

62 $$\begin{aligned} \dot{\hat{{\varvec{L}}}}_i^T = \begin{bmatrix} -\lambda \dot{x}_{3i} & 0 \\ 0 & -\lambda \dot{x}_{3i} \\ x_{1i} \dot{x}_{3i} + \dot{x}_{1i} x_{3i} & x_{2i} \dot{x}_{3i} + \dot{x}_{2i} x_{3i} \\ \lambda ^{-1}(\dot{x}_{1i}x_{2i} + x_{1i}\dot{x}_{2i}) & \lambda ^{-1}(2x_{2i}\dot{x}_{2i}) \\ -\lambda ^{-1}(2x_{1i}\dot{x}_{1i}) & -\lambda ^{-1}(\dot{x}_{1i}x_{2i} + x_{1i}\dot{x}_{2i}) \\ \dot{x}_{2i} & -\dot{x}_{1i} \end{bmatrix}. \end{aligned}$$

Additionally, the derivative of $\hat{P}_{ci}$ with respect to time can be expressed as follows:

63 $$\begin{aligned} \hat{P}_{ci} = \begin{bmatrix} \dot{x}_{1i} \\ \dot{x}_{2i} \\ \dot{x}_{3i} \end{bmatrix} = \begin{bmatrix} -\lambda x_{3i} & 0 & x_{1i}x_{3i} & \frac{x_{1i}x_{2i}}{\lambda } & -\frac{\lambda ^2 + x_{1i}^2}{\lambda } & x_{2i} \\ 0 & -\lambda x_{3i} & x_{2i}x_{3i} & \frac{\lambda ^2 + x_{2i}^2}{\lambda } & -\frac{x_{1i}x_{2i}}{\lambda } & -x_{1i} \\ 0 & 0 & x_{3i}^2 & \frac{x_{2i}x_{3i}}{\lambda } & -\frac{x_{1i}x_{3i}}{\lambda } & 0 \end{bmatrix} (V_c - V_m) \end{aligned}$$

It can be seen that $\dot{\hat{P}}_{ci} = [\dot{x}_{1i}, \dot{x}_{2i}, \dot{x}_{3i}]^{\textrm{T}}$ is a function of the visual servo controller (28). Therefore, combining equation (63) and the visual servo controller (28), we obtain as follows:

64 $$\begin{aligned} \begin{aligned} \Vert \dot{\hat{{\varvec{L}}}}\Vert&\le \sigma _d(\Vert {\varvec{e}}_s\Vert ) \cdot \Vert -k\hat{{\varvec{L}}}^{+}{\varvec{\varepsilon }} - {\varvec{e}}_m\Vert \\&\le \sigma _d(\Vert {\varvec{e}}_s\Vert ) \cdot \left( k\Vert {\varvec{e}}_L\Vert + \Vert {\varvec{e}}_m\Vert \right) \end{aligned} \end{aligned}$$

where $\sigma _d(\cdot )$ is another monotonically increasing function with respect to the image feature error $e_s$.

Furthermore, considering that within the set $\Omega _\xi$, due to the boundedness and time-convergence property of the performance function (22), ${\varvec{e}}_s$ is bounded for all $t \in [0, t_{\max })$. Therefore, for equations (59), (60), and (64), we have as follows:

65a $$\begin{aligned} \Vert \hat{{\varvec{L}}}\Vert&\le \sigma _h(\Vert {\varvec{e}}_s\Vert ) \le \overline{\sigma }_h , \end{aligned}$$

65b $$\begin{aligned} \Vert \hat{{\varvec{L}}}^+\Vert&\le \sigma _p(\Vert {\varvec{e}}_s\Vert ) \le \overline{\sigma }_p , \end{aligned}$$

65c $$\begin{aligned} \Vert \dot{\hat{{\varvec{L}}}}\Vert&\le \overline{\sigma }_d \cdot \left( k\Vert {\varvec{e}}_L\Vert + \Vert {\varvec{e}}_m\Vert \right) . \end{aligned}$$

According to the definition of equation (39), $\varphi _m$ takes effect if and only if $f(\hat{V}_m) > 0$ and $\phi _m^{\textrm{T}}\nabla f(\hat{V}_m) > 0$. According to reference^38^, when $\varphi _m$ satisfies the activation condition, $e_m^{\textrm{T}}\varphi _m \le 0$. Based on Assumption 1 and combining equation (54), rearranging equation (58) yields as follows:

66 $$\begin{aligned} \begin{aligned} \dot{V} \le&-c_3\Vert {\varvec{\varepsilon }}_L\Vert ^2 + 2c_2c_4\Vert {\varvec{e}}_m\Vert \Vert {\varvec{\varepsilon }}_L\Vert ^3 + 2c_2c_5\Vert {\varvec{e}}_m\Vert \Vert {\varvec{\varepsilon }}_L\Vert ^2 \\&- \frac{1}{\mu _2}\Vert {\varvec{e}}_m\Vert ^2 + \Vert {\varvec{e}}_m\Vert \overline{v}_{m2} + \frac{1}{\mu _2}\Vert {\varvec{e}}_m\Vert \Vert \hat{{\varvec{L}}}^+\Vert \Vert {\varvec{e}}_f\Vert \\&- \frac{1}{\mu _1}\Vert {\varvec{e}}_f\Vert ^2 + \Vert {\varvec{e}}_f\Vert \Vert \dot{\hat{{\varvec{L}}}}\Vert \overline{v}_{m1} + \Vert {\varvec{e}}_f\Vert \Vert \dot{\hat{{\varvec{L}}}}\Vert \overline{v}_{m2}. \end{aligned} \end{aligned}$$

Combining equation () and considering the definition and convergence property of the performance function (22), we obtain as follows:

67 $$\begin{aligned} \begin{aligned} \dot{V}&\le -c_3 \Vert {\varvec{\varepsilon }}_L\Vert ^2 + 2 c_2 c_4 \epsilon ^2 \Vert {\varvec{e}}_m\Vert \Vert {\varvec{\varepsilon }}_L\Vert + 2 c_2 c_5 \epsilon \Vert {\varvec{e}}_m\Vert \Vert {\varvec{\varepsilon }}_L\Vert \\&\quad - \frac{1}{\mu _2} \Vert {\varvec{e}}_m\Vert ^2 + \overline{v}_{m2} \Vert {\varvec{e}}_m\Vert + \frac{1}{\mu _2} \overline{\sigma }_p \Vert {\varvec{e}}_m\Vert \Vert {\varvec{e}}_f\Vert \\&\quad - \frac{1}{\mu _1} \Vert {\varvec{e}}_f\Vert ^2 + \overline{v}_{m1} \Vert {\varvec{e}}_f\Vert \overline{\sigma }_d\left( k \Vert {\varvec{\varepsilon }}_L\Vert + \Vert {\varvec{e}}_m\Vert \right) \\&\quad +\overline{\sigma }_h \overline{v}_{m2} \Vert {\varvec{e}}_f\Vert , \quad \forall \Vert {\varvec{\varepsilon }}_L\Vert < \epsilon \end{aligned} \end{aligned}$$

where $\epsilon$ is a constant that satisfies as follows:

$$\begin{aligned} 0< \epsilon < \min \left\{ r, \left( -\frac{c_5}{2c_4} + \sqrt{\frac{c_5^2}{4c_4^2} + \frac{c_3}{c_2c_4}} \right) \right\} . \end{aligned}$$

Using the properties of Young’s inequality, we get as follows:

$$\begin{aligned} 2c_2c_4\epsilon ^2\Vert {\varvec{e}}_m\Vert \Vert {\varvec{e}}_L\Vert&\le \varpi _1\Vert {\varvec{e}}_m\Vert ^2 + \varpi _1\Vert {\varvec{e}}_L\Vert ^2 \\ 2c_2c_5\epsilon \Vert {\varvec{e}}_m\Vert \Vert {\varvec{e}}_L\Vert&\le \varpi _2\Vert {\varvec{e}}_m\Vert ^2 + \varpi _2\Vert {\varvec{e}}_L\Vert ^2 \\ \overline{v}_{m2}\Vert {\varvec{e}}_m\Vert&\le \frac{\kappa _1}{2}\Vert {\varvec{e}}_m\Vert ^2 + \frac{\overline{v}_{m2}^2}{2\kappa _1} \\ \frac{1}{\mu _2}\overline{\sigma }_p\Vert {\varvec{e}}_m\Vert \Vert {\varvec{e}}_f\Vert&\le \frac{\overline{\sigma }_p}{2\mu _2}\Vert {\varvec{e}}_m\Vert ^2 + \frac{\overline{\sigma }_p}{2\mu _2}\Vert {\varvec{e}}_f\Vert ^2 \\ \overline{v}_{m1}\overline{\sigma }_d k\Vert {\varvec{e}}_f\Vert \Vert {\varvec{e}}_L\Vert&\le \frac{k\varpi _3}{2}\Vert {\varvec{e}}_L\Vert ^2 + \frac{k\varpi _3}{2}\Vert {\varvec{e}}_f\Vert ^2 \\ \overline{v}_{m1}\overline{\sigma }_d\Vert {\varvec{e}}_f\Vert \Vert {\varvec{e}}_m\Vert&\le \frac{\varpi _3}{2}\Vert {\varvec{e}}_m\Vert ^2 + \frac{\varpi _3}{2}\Vert {\varvec{e}}_f\Vert ^2 \\ \overline{\sigma }_h\overline{v}_{m2}\Vert {\varvec{e}}_f\Vert&\le \frac{\kappa _2}{2}\Vert {\varvec{e}}_f\Vert ^2 + \frac{\overline{\sigma }_h^2\overline{v}_{m2}^2}{2\kappa _2}. \end{aligned}$$

Substituting the above inequalities into equation (67) gives as follows:

68 $$\begin{aligned} \begin{aligned} \dot{V} \le&-c_3 \Vert {\varvec{e}}_L\Vert ^2 + \varpi _1 \Vert {\varvec{e}}_L\Vert ^2 + \varpi _2 \Vert {\varvec{e}}_L\Vert ^2 \\&+ \frac{k \varpi _3}{2} \Vert {\varvec{e}}_L\Vert ^2- \frac{1}{\mu _2} \Vert {\varvec{e}}_m\Vert ^2 + \varpi _1 \Vert {\varvec{e}}_m\Vert ^2 \\&+ \varpi _2 \Vert {\varvec{e}}_m\Vert ^2 + \frac{\kappa _1}{2} \Vert {\varvec{e}}_m\Vert ^2 + \frac{\overline{\sigma }_p}{2 \mu _2} \Vert {\varvec{e}}_m\Vert ^2 \\&+ \frac{\varpi _3}{2} \Vert {\varvec{e}}_m\Vert ^2 - \frac{1}{\mu _1} \Vert {\varvec{e}}_f\Vert ^2 + \frac{\overline{\sigma }_p}{2 \mu _2} \Vert {\varvec{e}}_f\Vert ^2 \\&+ \frac{k \varpi _3}{2} \Vert {\varvec{e}}_f\Vert ^2 + \frac{\varpi _3}{2} \Vert {\varvec{e}}_f\Vert ^2+ \frac{\kappa _2}{2} \Vert {\varvec{e}}_f\Vert ^2 \\&+ \frac{\overline{v}_{m2}^2}{2 \kappa _1} + \frac{\overline{\sigma }_h^2 \overline{v}_{m2}^2}{2 \kappa _2}. \end{aligned} \end{aligned}$$

By selecting sufficiently large control gain *k*, sufficiently small filter time constants $\mu _1$ and $\mu _2$, and appropriate parameters $\kappa _1$ and $\kappa _2$, the inequalities hold as follows:

69 $$\begin{aligned} \rho _1&= c_3 - \varpi _1 - \varpi _2 - \frac{k \varpi _3}{2} > 0 , \end{aligned}$$

70 $$\begin{aligned} \rho _2&= \frac{1}{\mu _2} - \varpi _1 - \varpi _2 - \frac{\kappa _1}{2} - \frac{\overline{\sigma }_p}{2\mu _2} - \frac{\varpi _3}{2} > 0 ,\end{aligned}$$

71 $$\begin{aligned} \rho _3&= \frac{1}{\mu _1} - \frac{\overline{\sigma }_p}{2\mu _2} - \frac{(k+1)\varpi _3}{2} - \frac{\kappa _2}{2} > 0. \end{aligned}$$

Rearranging equation (68) yields as follows:

72 $$\begin{aligned} \dot{V} \le -\rho V + \delta \end{aligned}$$

where

73 $$\begin{aligned} \begin{aligned} \delta&= \frac{\overline{v}_{m2}^2}{2\kappa _1} + \frac{\overline{\sigma }_h^2 \overline{v}_{m2}^2}{2\kappa _2}, \\ \rho&= \min \left\{ \rho _1 / c_2, 2\rho _2, 2\rho _3 \right\} . \end{aligned} \end{aligned}$$

For equation (72), when $V = \varsigma$ and $\rho > \delta /\varsigma$, $\dot{V} < 0$. Thus, if $V(0) \le \varsigma$ and parameters satisfy $\rho > \delta /\varsigma$, then $V \le \varsigma$ for all $t \in [0, \tau _{\max })$. Solving equation (72) gives as follows:

74 $$\begin{aligned} V(t) \le V(0) \exp (-\rho t) + \varrho \le V(0) + \varrho , \quad \forall t \in [0, \tau _{\max }) \end{aligned}$$

with $\varrho = \delta / \rho$. From equations (56) and (74), as $t \rightarrow \tau _{\max }$:

$$\begin{aligned} \Vert \varepsilon _L \Vert&\le \sqrt{\varrho / c_2}< \epsilon , \\ \Vert e_m \Vert&< \sqrt{2\varrho }, \\ \Vert e_f \Vert&\le \sqrt{2\varrho }. \end{aligned}$$

Thus, $\varepsilon _L$, $e_m$, and $e_f$ converge to neighborhoods of zero. The radii $\sqrt{\varrho / c_2}$ and $\sqrt{2\varrho }$ can be made arbitrarily small via parameter adjustment. When $\hat{L}^+$ has full row rank, $\Vert \epsilon _L\Vert \triangleq \Vert \hat{L}^+\epsilon \Vert \ne 0$ for $\epsilon \ne 0$ near $\epsilon _L = 0$^39^. Hence, $\Vert e\Vert \le \varepsilon$ for some $\varepsilon > 0$, confirming $\epsilon$ converges to a $\overline{\varepsilon }$-neighborhood of zero. From equations (26a) and (26b) as follows:

75 $$\begin{aligned} \begin{aligned} -\underline{e}_{ui,0}&< -\underline{e}_{ui,0} \left( \frac{ \exp (\overline{\varepsilon }/k_{ui}) - \overline{e}_{ui,0}/\underline{e}_{ui,0} }{ \exp (\overline{\varepsilon }/k_{ui}) + 1 } \right) \le \xi _{ui}< \overline{e}_{ui,0} \\ -\underline{e}_{vi,0}&< -\underline{e}_{vi,0} \left( \frac{ \exp (\overline{\varepsilon }/k_{vi}) - \overline{e}_{vi,0}/\underline{e}_{vi,0} }{ \exp (\overline{\varepsilon }/k_{vi}) + 1 } \right) \le \xi _{vi} < \overline{e}_{vi,0}. \end{aligned} \end{aligned}$$

Combining equation (75) with constraints (19)-(21), the pixel coordinate error $e_s$ is bounded for $t \in [0, \tau _{\max })$ with guaranteed transient and steady-state performance. Thus, equation (21) holds, and backstepping confirms satisfaction of equation (19), ensuring persistent FOV compliance.

Since equation (19) holds, feature pixel $s_i = [u_i, v_i]^T$ is bounded. By Assumption 1 and bounded $e_m$, velocity estimate $\hat{v}_m$ is bounded. Controller (28) yields bounded $v_c$, so UAV velocity input (43) and gimbal rate (45) are bounded. Thus, all closed-loop signals are ultimately bounded.

In summary, control input (28) satisfies $t \in [0, \tau _{\max })$. Inequalities (75) imply $(\xi , t)$ remains in compact set $\Omega '_\xi \subset \Omega _\xi$. Letting $\partial \Omega _\xi$ be the boundary, there exists $d > 0$ such that:

$$d_S((s, t), \partial \Omega _\xi ) \ge d, \quad \forall t \in [0, \tau _{\max }).$$

Hence,

$$\lim _{t \rightarrow \tau _{\max }} \left( \Vert s(t) \Vert + \left[ d_{\mathcal {S}}((s, t), \partial \Omega _\xi ) \right] ^{-1} \right) \ne \infty .$$

By Lemma^40^ and contradiction, $\tau _{\max } = \infty$.

## Simulation Results

**Fig. 5:**
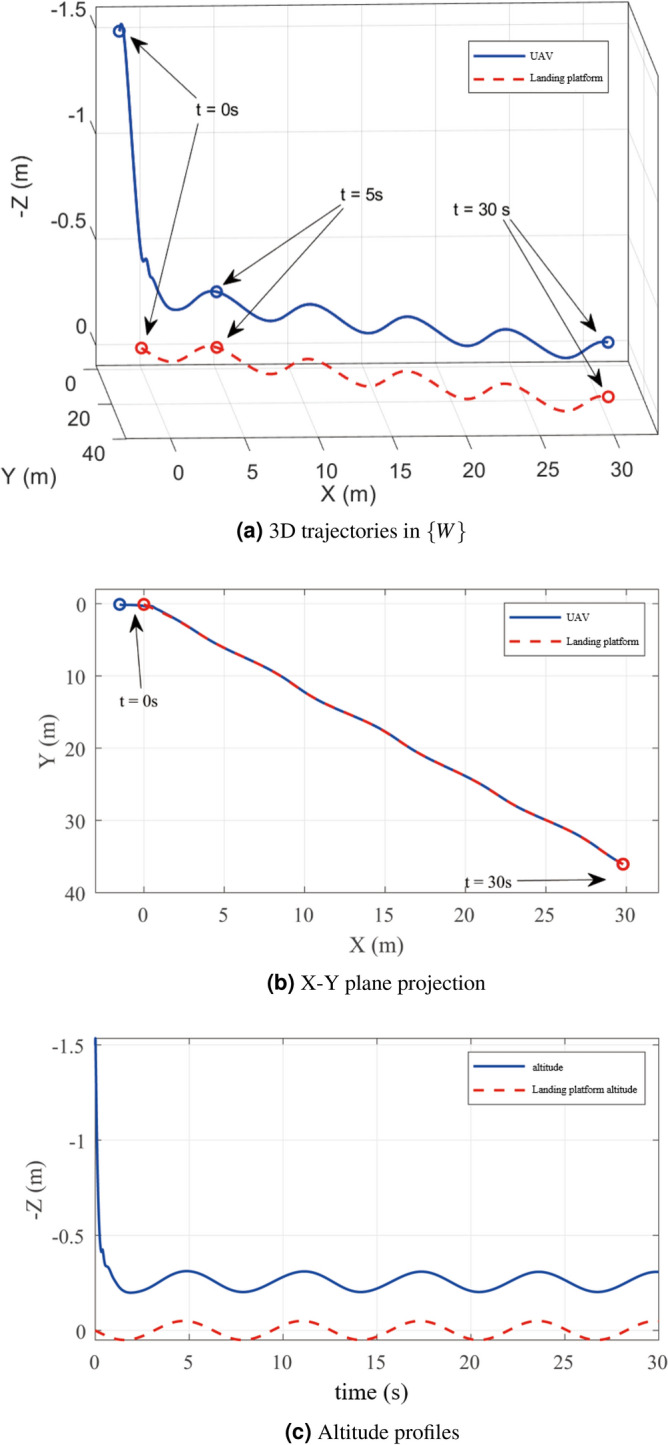
Trajectory comparison: (**a**) 3D trajectories in $\{W\}$, (**b**) X–Y plane projection, (**c**) Altitude profiles.

This section presents simulation studies validating the effectiveness of the proposed control algorithm. The UAV parameters are configured as follows: mass $m = 2.48 \text {kg}$, inertia matrix $J = \text {diag}\{0.0756, 0.0756, 0.1277\} \text {kg}\cdot \text {m}^2$, and gravitational acceleration $g = 9.81 \text {m/s}^2$. The camera features a focal length $\lambda = 376.1587$ and a pixel resolution of $640 \times 480$. The position controller employs the backstepping method for velocity input (43), while the attitude controller follows^41^ specifications. Based on equation (19), the camera FOV-constraints define the permissible feature point movement range in pixels as follows:

76a $$\begin{aligned} u_{\max }&= 319,&u_{\min }&= -319, \end{aligned}$$

76b $$\begin{aligned} v_{\max }&= 239,&v_{\min }&= -239. \end{aligned}$$

Four image feature points are uniformly distributed at the corners of a 20 cm square, with the landing platform center coinciding with their centroid. The desired pixel coordinates $\textbf{s}_{di} = [u_{di}, v_{di}]^T$ are as follows:

$$\begin{aligned} \textbf{s}_{d1}&= [-150, 150]^T,&\textbf{s}_{d2}&= [150, 150]^T, \\ \textbf{s}_{d3}&= [-150, -150]^T,&\textbf{s}_{d4}&= [150, -150]^T, \end{aligned}$$

yielding $\textbf{s}_d = [\textbf{s}_{d1}^T, \textbf{s}_{d2}^T, \textbf{s}_{d3}^T, \textbf{s}_{d4}^T]^T \in \mathbb {R}^8$. According to equation (20), the initial error bounds for the integrated feature point coordinates are as follows:

$$\begin{aligned} \overline{\textbf{e}}_{u,0}&= [169, 469, 169, 469]^T,&\underline{\textbf{e}}_{u,0}&= [469, 169, 469, 169]^T ,\\ \overline{\textbf{e}}_{v,0}&= [389, 389, 89, 89]^T,&\underline{\textbf{e}}_{v,0}&= [89, 89, 389, 389]^T. \end{aligned}$$

The performance function parameters are set as $(e_{ui,\infty }, e_{vi,\infty }) = (30, 30)$ and $(\kappa _{ui}, \kappa _{vi}) = (0.2, 0.2)$, with control gain $k = 5$. Initial states include the gimbal orientation $\varvec{\eta }_{g0} = [0, \pi /5, \pi /12]^T$ rad and UAV state as follows:

$$\begin{aligned} \textbf{p}_0&= [-1.5, 0.1, -1.5]^T ,\\ \textbf{v}_0&= [0, 0, 0]^T, \\ R_0&= I ,\\ \varvec{\Omega }_0&= [0, 0, 0]^T. \end{aligned}$$

The landing platform trajectory in world frame $\{W\}$ is governed as follows:

$$\begin{aligned} x_t&= 1.0t + 0.2\sin (t), \\ y_t&= 1.2t + 0.1\cos (0.6t) ,\\ z_t&= 0.15\sin (t). \end{aligned}$$

**Fig. 6:**
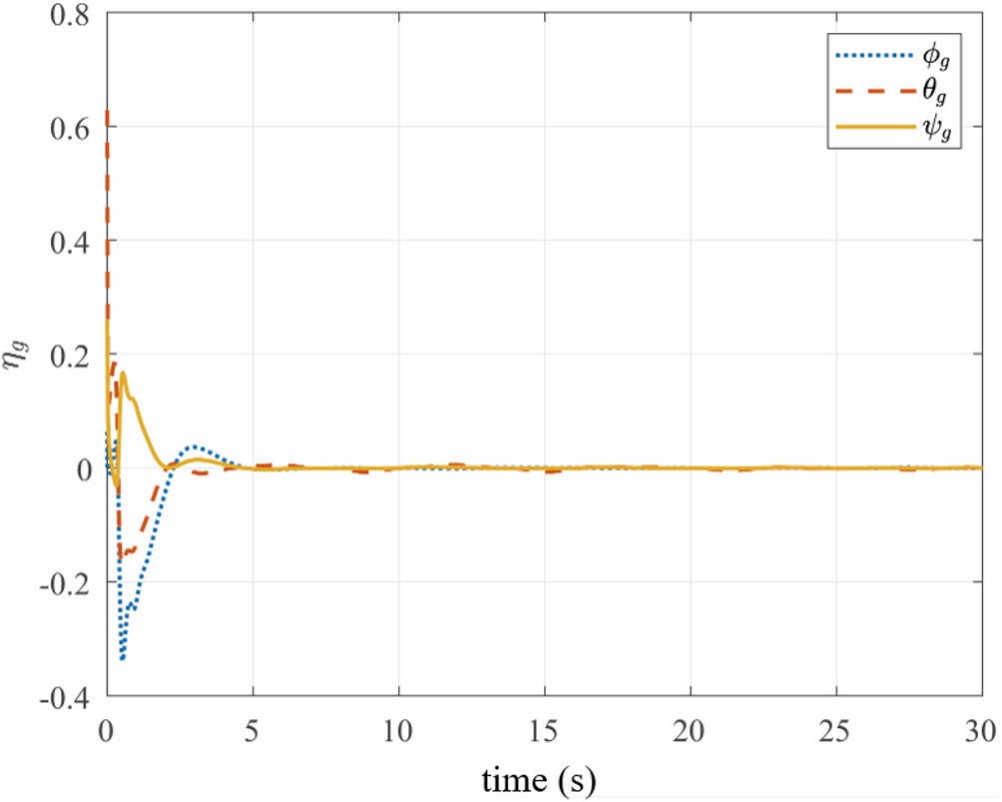
Gimbal attitude evolution during landing maneuver.

**Fig. 7:**
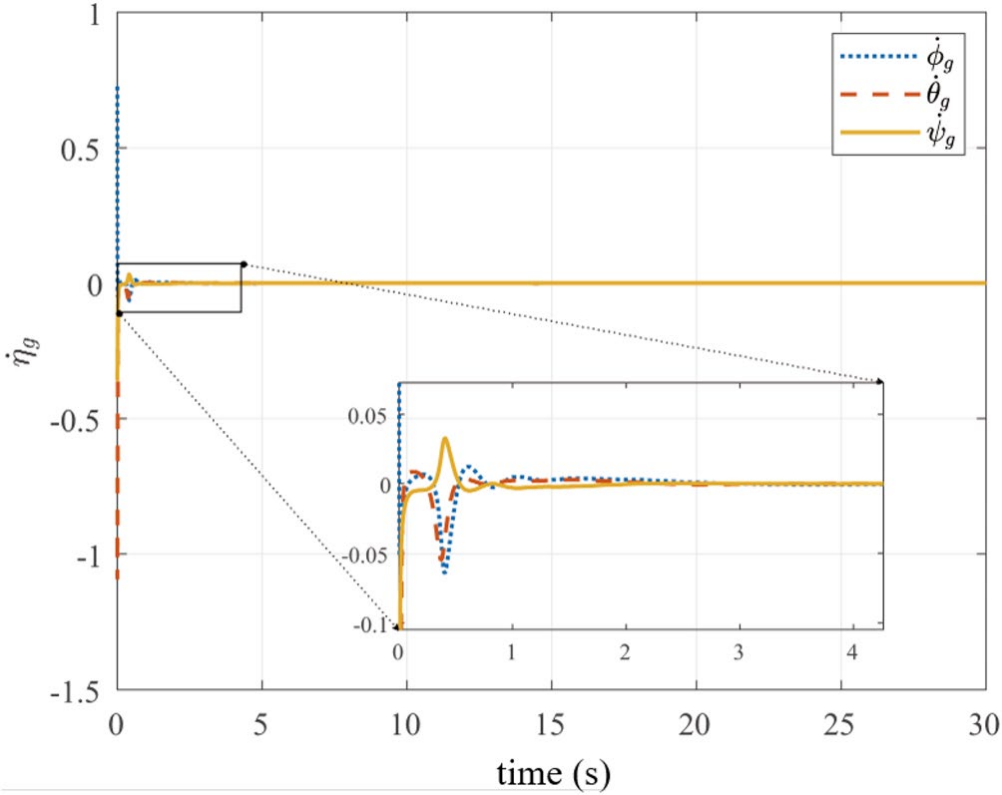
Gimbal angular velocities.

**Fig. 8:**
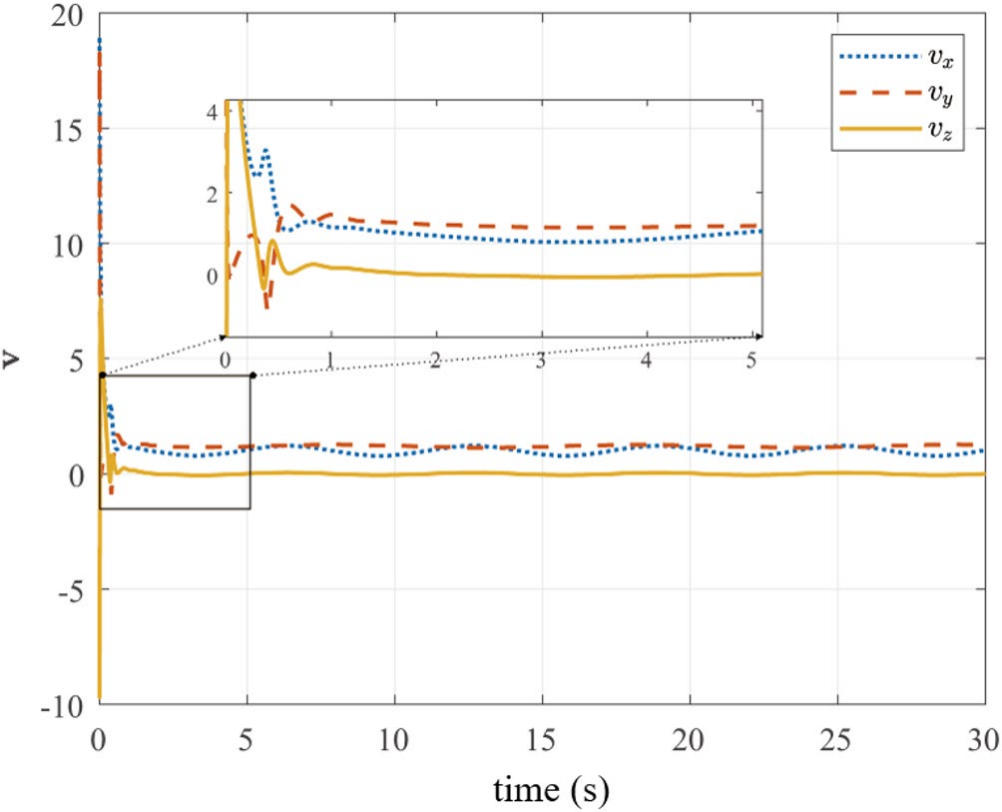
UAV translational velocities.

**Fig. 9:**
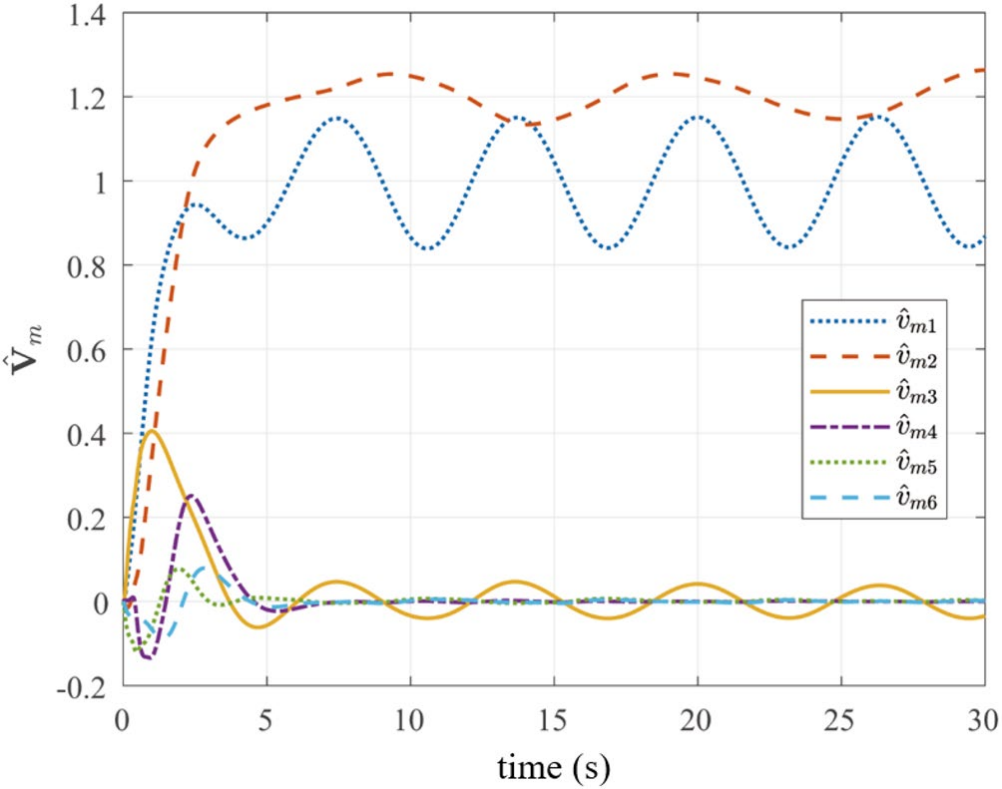
Landing platform velocity estimation.

Figure 5 illustrates the 3D motion trajectories. The UAV trajectory (solid blue line) and platform path (red dashed line) are shown in Fig. 5a, with markers indicating positions at key timepoints (0 s, 5 s, 30 s).

Figure 5b shows the X-Y plane projection. Initial position offset at $t = 0$ s is eliminated by $t = 5$ s, after which precise trajectory tracking is maintained. Figure 5c demonstrates stable altitude regulation despite vertical platform oscillations, confirming the controller’s disturbance rejection capability.

Fig. 6 presents gimbal attitude convergence from $\varvec{\eta }_{g0}$ to $[0, 0, 0]^T$ rad within 5 s. Angular velocities (Fig. 7) and UAV linear velocities (Figure 8) exhibit smooth transitions with convergence near zero, validating the physical realizability of control commands from (28).

Figure 9 depicts the estimated landing platform velocity $\hat{\varvec{V}}_m$. The estimator achieves stable velocity reconstruction within 5 s, with errors converging to near-zero values, demonstrating the effectiveness of the velocity estimation method for unknown moving targets described in before.

**Fig. 10:**
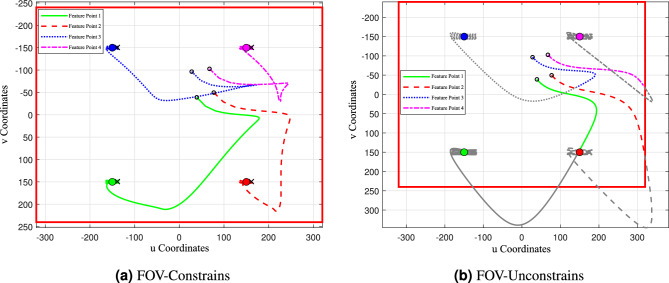
A comparative analysis of feature point pixel trajectories under FOV-constraints.

### Comparative simulations under FOV-constraints

To evaluate the effectiveness of incorporating camera FOV-constraints, a comparative simulation is conducted between the proposed landing algorithm and the conventional IBVS approach reported in paper ^36^. The corresponding results are presented in Figure 10.

Figure 10adepicts the pixel trajectories of the feature points, illustrating the evolution of image feature errors under the two controllers. In these plots, the circles near the center represent the initial positions of the feature points, the black crosses denote their final positions at the end of the simulation, and the solid colored circles indicate their desired pixel locations.

As shown in Figure 10a, under the proposed FOV-constraints controller, all four feature points remain within the camera’s visible range (demarcated by the red bounding box) and eventually converge to the vicinity of their desired positions. In contrast, Figure 10bpresents the trajectories generated by the conventional IBVS method. The trajectory segments where feature points exit the FOV are highlighted in grey. Although the feature points eventually converge close to their desired positions, in practice, once a feature point leaves the FOV, it becomes untrackable. Compared with Figure 10a, the unconstrained controller produces larger oscillations in the pixel space.

**Fig. 11:**
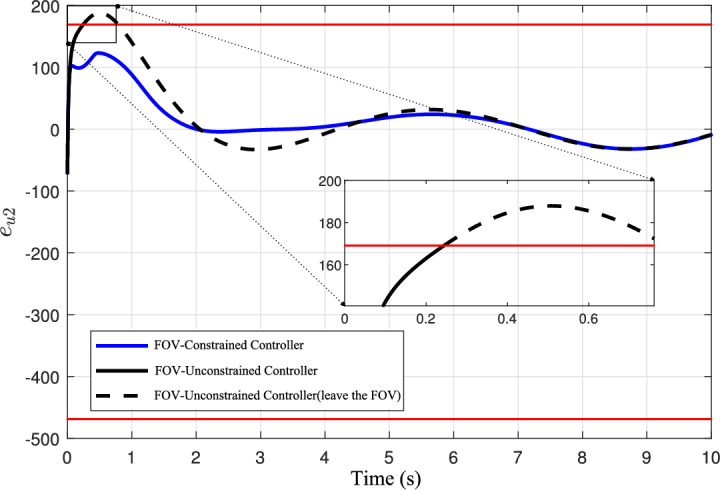
Block diagram of vision-constrained gimbal-servo UAV landing control system.

**Fig. 12:**
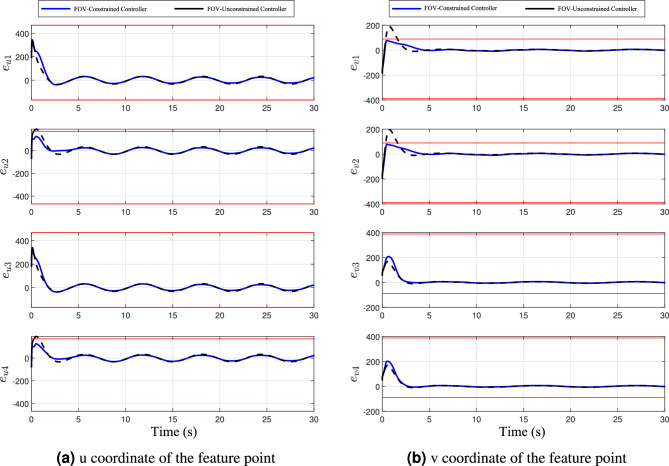
A comparative analysis of feature point pixel errors under FOV-constraints.

A closer examination reveals that Feature Point 2 is the first to leave the FOV, as observed in Fig. 10. To further analyze this case, Figure 11 illustrates the evolution of its *u* coordinate error $e_{u2}$. The proposed method is shown in blue, while the conventional algorithm is shown in black. The FOV error bound is represented by a solid red line, and the region beyond the visible range is marked with a black dashed line. It can be seen that the proposed method ensures that the pixel error of Feature Point 2 always remains within the FOV, whereas the conventional IBVS method results in the error exceeding the FOV boundary at *t* = 0 s.

Furthermore, Figure  12 presents the evolution of the overall pixel error $e_s$. These results confirm that, in the absence of FOV-constraints, feature points are prone to leave the visible range, which not only causes a loss of tracking but may also lead to larger convergence errors. By contrast, the proposed FOV-constraints algorithm effectively guarantees that all feature points remain observable throughout the landing process, thereby enhancing the robustness and reliability of visual servo control.

## Conclusion

This paper presented a vision-constrained gimbal-servo landing control algorithm for UAVs, designed to address the limitations of fixed-camera approaches in autonomous landing. By incorporating FOV constraints and exploiting the agility of a three-axis gimbal, the proposed controller ensures persistent visibility of image features throughout the descent. A prescribed performance-based framework was employed to rigorously regulate the transient and steady-state behavior of image feature errors, while a velocity observer was introduced to estimate platform motion in real time, enhancing robustness against unknown disturbances. Simulation studies demonstrated that the proposed method enables the UAV to achieve precise landing on a moving platform. The results confirmed that image feature errors and velocity estimation errors converge rapidly, the gimbal and UAV velocities remain smooth and physically realizable, and the landing trajectory closely aligns with the motion of the platform. Comparative analysis further highlighted that, unlike unconstrained IBVS controllers, the proposed approach successfully prevents feature points from leaving the FOV, thereby guaranteeing reliable tracking and significantly reducing oscillations in the image plane. Overall, the proposed algorithm provides a robust and adaptive framework for UAV autonomous landing under visual constraints. Its effectiveness in simulation suggests strong potential for real-world deployment, particularly in dynamic and unstructured environments. Future work will extend this study to hardware-in-the-loop experiments and outdoor flight tests, as well as investigate the integration of external disturbance rejection mechanisms to enhance adaptability under challenging environmental conditions.

## Data Availability

The data presented in this study are available on request from the corresponding author.
